# Measurement of Glutathione as a Tool for Oxidative Stress Studies by High Performance Liquid Chromatography

**DOI:** 10.3390/molecules25184196

**Published:** 2020-09-13

**Authors:** Faisal Nuhu, Andrew Gordon, Roger Sturmey, Anne-Marie Seymour, Sunil Bhandari

**Affiliations:** 1 Centre for Atherothrombosis and Metabolic Disease, Hull York Medical School, University of Hull, Hull HU6 7RX, UK; fisafun@yahoo.co.uk (F.N.); Andrew.gordon@hyms.ac.uk (A.G.); Roger.sturmey@hyms.ac.uk (R.S.); Andi.Seymour.19@gmail.com (A.-M.S.); 2 Hull York Medical School & Department of Renal Medicine, Hull University Teaching Hospitals Trust, Anlaby Road, Kingston upon, Hull HU3 2JZ, UK

**Keywords:** glutathione analysis, oxidative stress, HPLC, GSH, GSSG

## Abstract

Background: Maintenance of the ratio of glutathione in the reduced (GSH) and oxidised (GSSG) state in cells is important in redox control, signal transduction and gene regulation, factors that are altered in many diseases. The accurate and reliable determination of GSH and GSSG simultaneously is a useful tool for oxidative stress determination. Measurement is limited primarily to the underestimation of GSH and overestimation GSSG as a result of auto-oxidation of GSH. The aim of this study was to overcome this limitation and develop, optimise and validate a reverse-phase high performance liquid chromatographic (HPLC) assay of GSH and GSSG for the determination of oxidant status in cardiac and chronic kidney diseases. Methods: Fluorescence detection of the derivative, glutathione-O-pthaldialdehyde (OPA) adduct was used. The assay was validated by measuring the stability of glutathione and glutathione-OPA adduct under conditions that could affect the reproducibility including reaction time and temperature. Linearity, concentration range, limit of detection (LOD), limit of quantification (LOQ), recovery and extraction efficiency and selectivity of the method were assessed. Results: There was excellent linearity for GSH (r^2^ = 0.998) and GSSG (r^2^ = 0.996) over concentration ranges of 0.1 µM–4 mM and 0.2 µM–0.4 mM respectively. The extraction of GSH from tissues was consistent and precise. The limit of detection for GSH and GSSG were 0.34 µM and 0.26 µM respectively whilst their limits of quantification were 1.14 µM and 0.88 µM respectively. Conclusion: These data validate a method for the simultaneous measurement of GSH and GSSG in samples extracted from biological tissues and offer a simple determination of redox status in clinical samples.

## 1. Introduction

**Figure 1:**
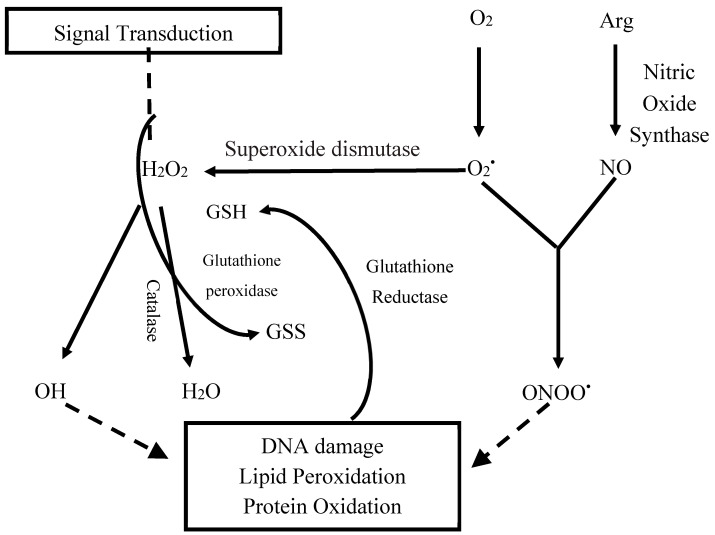
Reactive oxygen species (ROS) generation and effect in a cardiomyocyte. It begins with partial reduction of oxygen (O_2_) to give superoxide (O_2_^•^), ultimately generating hydroxyl radical (OH^•^). ONOO^•^ (peroxynitrite), hydrogen peroxide (H_2_O_2_), arginine (Arg), nitric oxide (NO), oxidised glutathione GSSG, reduced glutathione GSH, water (H_2_O).

Oxidative stress is defined as the imbalance between reactive oxygen species (ROS) production and antioxidant defences in a biological system [1]. ROS are unstable and highly reactive oxygen species with a single electron and an unpaired electron in the outer orbit. ROS including superoxide anions (O_2_^•^) and hydroxyl radicals (OH^•^) hydrogen peroxide (H_2_O_2_) are produced by endothelial cells, inflammatory cells and the mitochondrial electron transport system [2]. The production of ROS radicals (Figure 1) starts by the one electron reduction of oxygen (O_2_) to O_2_^•^ which under the influence of the enzyme superoxide dismutase is converted to H_2_O_2_. These two ROS then form the basis for the generation of further and even more dangerous radicals.

The glutathione system consisting of glutathione reductase, glutathione oxidase and glutathione, maintains the concentration of O_2_^•^ and H_2_O_2_ at physiological levels necessary for tissue repair and immune defence [3]. Therefore, the ratio of oxidised and reduced glutathione indicates the redox state of a cell and may represent a valuable tool for the assessment of oxidative stress and a target for drug-based antioxidant therapies [4]. Under chronic pathological conditions, compromised or ineffective antioxidant capacity including the glutathione system results in excess ROS generation. Consequences of this include oxidative damage to DNA, proteins and cell membrane lipids, and altered cellular signal transductions as observed in many disorders such as diabetes, cardiovascular, autoimmune and chronic kidney diseases [5]. The pathway of systemic and tissue level oxidative stress in these diseases is complex leading to inflammation and subsequent effects on vascular remodelling, endothelial function and mitochondrial dysfunction [6,7].

Glutathione (GSH) is a ubiquitous thiol tripeptide (γ-l-glutamyl-l-cysteinyl-glycine) found in a range of cells including cardiomyocytes [8], hepatocytes and erythrocytes [9]. The majority of GSH (90%) is found in the cytosol with a small but significant amount in the mitochondria and endoplasmic reticulum (ER) [10,11,12]. GSH is synthesised de novo from glutamate, cysteine and glycine [13] with a γ-carboxyl linkage between glutamate and cysteine of GSH, which is only susceptible to hydrolysis by γ glutamyltranspeptidase, [14] rather than the usual α-carboxyl group. This confers resistance to intracellular degradation. GSH plays a number of important biological functions including scavenging oxygen-derived free radicals to ease oxidative stress [15,16]; signalling in apoptosis [17,18]; modulating cellular process such as immune response and DNA synthesis [8]; detoxifying electrophiles [19]; and acting as cysteine reservoir [20]. The role as an oxygen radical scavenging molecule is the current best characterised role of GSH.

Mitochondrial respiration is crucial for cellular function, but it is also an endogenous source of ROS. The flow of electrons through the electron transport chain (ETC) can generate superoxide radicals (O_2_^•^) at Complexes I and II through the partial reduction of O_2_ [21,22]. Under normal conditions, this O_2_^•^ is cleared to hydrogen peroxide (H_2_O_2_) by superoxide dismutase (SOD) in the mitochondrial matrix [23]. However, H_2_O_2_ can also lead to the production of the more damaging oxygen radical, OH^•^, which can initiate a cascade of cellular injury through peroxidation of lipid, protein and DNA [24]. A key role of GSH is to prevent this damage by reducing H_2_O_2_ to water (H_2_O) in the presence of selenium-dependent glutathione peroxidase, via conversion of GSH to GSSG. In turn GSSG may be recycled to GSH by glutathione reductase with NADPH as the electron donor.

Mitochondrial GSH homeostasis is particularly important because, under pathological conditions, excessive generation of ROS results in toxic accumulation of GSSG [25]. This can lead to an imbalance in the redox potential, export of GSSG and eventual depletion of GSH, a classical state of oxidative stress [26] observed in a number of diseases including inflammation [27], autoimmune disorders [28,29] and liver disorders [30]. Hence, the reliable determination of GSH and GSSG in absolute and ratiometric terms in blood and tissues is regarded a putative tool in oxidative stress studies.

Several methods have been reported for measuring glutathione based on ultra-violet (UV) absorbance, fluorescence, spectrophotometry, electrochemical and tandem mass spectroscopy, usually after protein precipitation in biological samples [31,32,33,34,35,36], all of which have limitations. A recent excellent review has highlighted the ongoing challenges in using GSH/GSSG titration as a measure of thiol redox balance and the best pre-analytical and analytical methods for the quantification of these molecules in biological samples [37]. High performance liquid chromatography (HPLC) techniques with UV detection have poor limits of detection [38], restricting widespread application. HPLC methods with either UV or fluorescence detection require derivatisation of GSH prior to detection which causes further limitations driven principally as a result of enhanced susceptibility to GSH degradation and auto oxidation, instability of derivatised GSH adduct and suboptimal derivatisation conditions. The use of HPLC with electrochemical detection can overcome these problems by direct measurement of GSH and GSSG [39,40] but can be very expensive. Thus, the search for a fast, sensitive and reliable method for glutathione quantification is still ongoing. Kander et al. [31] developed an HPLC method for the quantification of GSH and GSSG in human plasma and whole blood using metaphosphoric acid as a precipitant. Their method showed reduction of GSH by 25% within one hour of sample collection, potentially due to auto-oxidation and/or degradation. We present a modified version of the method developed by Kander et al. [31] and we have further optimised conditions to minimise the problems of auto-oxidation and degradation for the study of oxidative stress in biological tissues.

The objective of this study was to characterise the stability of GSH and its adduct with O-pthaldialdehyde (OPA) and validate an optimised, rapid and accurate method for the simultaneous measurement of GSH and GSSG for oxidative stress studies using reverse phase HPLC in cardiac, skeletal, liver and kidney tissues.

## 2. Results

**Figure 2:**
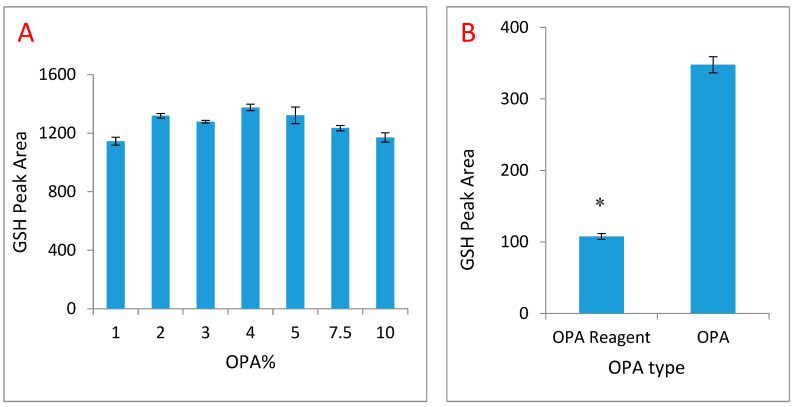
(**A**) Summary figure of the optimisation of glutathione-O-pthaldialdehyde (OPA) concentration using a 2000μM reduced glutathione (GSH) standard. (**B**) Reactivity of OPA and OPA reagent with 400 μM GSH standard. Data are presented as mean ± (standard error of mean (SEM) (*n* = 5, * *p* < 0.05). Standards prepared using N-ethylmaleimide (NEM) and incubated for up to 10 min and assayed by high performance liquid chromatography (HPLC). Peak areas at a wavelength of 450 nm were measured.

O-pthaldialdehyde (OPA) optimisation: Method optimisation identified that a concentration of purified OPA between 1% and 5% (*v*/*v*) overcame complications of interference but with sufficient reactivity (Figure 2A). Furthermore, OPA reagent reacted with GSH leading to detection of 68.2% of total glutathione in 400 µmM GSH relative to pure (≥99.0%) OPA (Figure 2B).

### 2.1. Time and Temperature Optimisation

**Figure 3:**
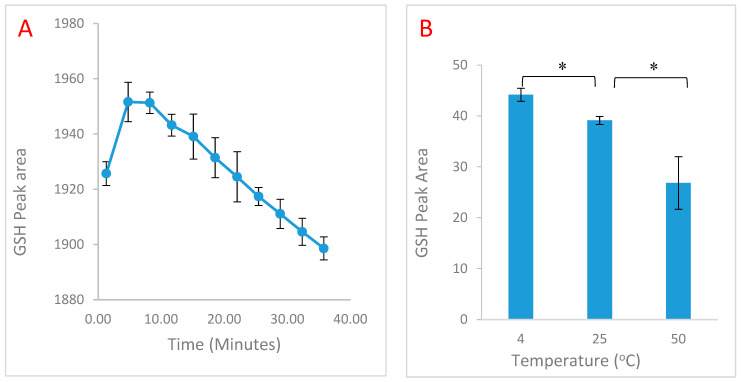
(**A**) Optimal incubation time required of 4 mM reduced glutathione (GSH) with 5% O-pthaldialdehyde (OPA) and subsequent degradation. (**B**) 200μM GSH run at different assay temperatures. Data are presented as mean ± standard error of mean (SEM) (*n* = 5, * *p* < 0.05).

Since the GSH-OPA adduct may be susceptible to time- and temperature-dependent spontaneous degradation, the incubation times were increased to prevent underestimation of GSH in biological samples. As shown in Figure 2, incubation of GSH and OPA at 25 °C generated better reactivity but when prolonged, resulted in time-dependent degradation (Figure 3A). Incubation time of 5–10 min was sufficient for optimal reaction with limited degradation. To confirm the temperature-dependent stability of the GSH-OPA adduct, the assay was performed at 4 °C, 25 °C and 50 °C (Figure 3B). These data show that the GSH-OPA adduct was most stable at 4 °C and degraded in a temperature-dependent fashion. 

### 2.2. NEM Optimisation and Autoxidation

**Figure 4:**
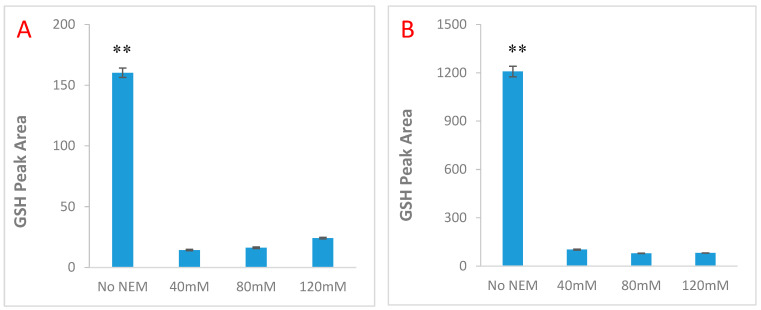
N-ethylmaleimide (NEM) optimisation using different amount of GSH (**A**) 200 μM (**B**) 2000 μM. The results also showed that 10–15% of GSH is oxidised to GSSG. Data are presented as mean ± SEM (*n* = 5, ** *p* < 0.05).

**Figure 5:**
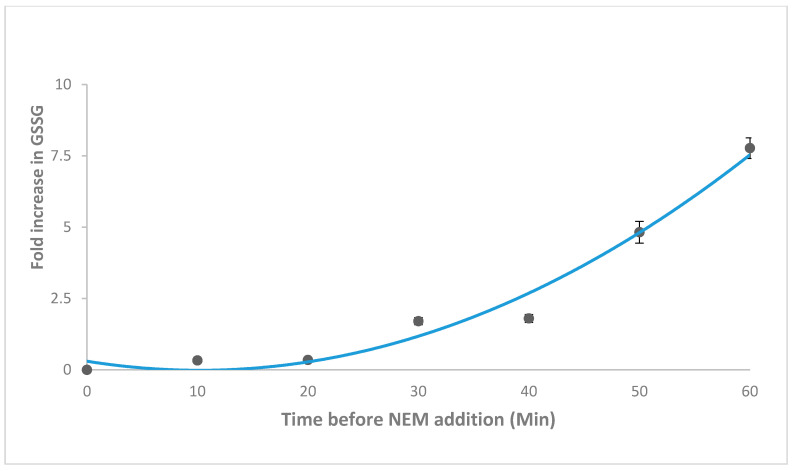
Time dependent auto-oxidation of reduced glutathione (GSH) with fold increase in oxidised glutathione (GSSG). 200 uM GSH was subjected to GSSG assay protocol at different incubation times. Samples were incubated at 4 °C for varying times, 40 mM NEM was added and incubated with O-pthaldialdehyde (OPA) for 25 min. Data are presented as mean ± SEM (*n* = 3).

The results of *N*-ethylmaleimide (NEM) optimisation indicated that 40 mM NEM was sufficient to conjugate GSH within the limit of the method (200–2000 μM). The data also showed that 10–15% of GSH was oxidised to GSSG (Figure 4A,B), thus sample preparation must be expedient. As a result, the time-dependent auto-oxidation of 200 µM GSH was ascertained as shown in Figure 5. The auto-oxidation experiment was also conducted to determine the impact of temperature. The results showed that at temperatures greater than 20 °C, GSH was degraded rather than auto-oxidised as indicated by the reductions in level of GSSG by 15% and 67% and consequently decrements in the GSSG/GSH ratio by 13% and 61% at 25 °C and 50 °C relative to 4 °C. In summary the GS-NEM adduct was found to be stable under the conditions of the assay (temperature, time and concentration).

### 2.3. Stability of Glutathione Extract

**Figure 6:**
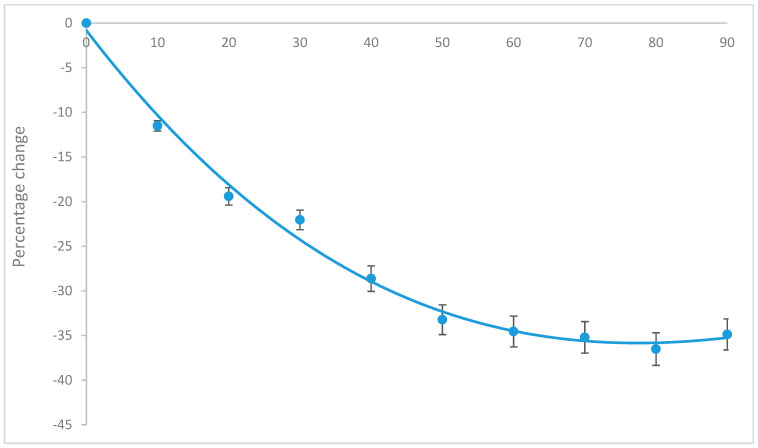
Time-dependent change of the degradation of reduced glutathione (GSH) from extracted cardiac tissue over time at 4 °C. Data are presented as mean ± SEM (*n* = 3).

Glutathione extracted from biological samples (cardiac, liver, whole blood or serum) may contain other substances, including trace metals, which may interact with, and compromise the stability of GSH. To explore the extent to which GSH measurement may be modified in biological samples, PCA extract from 200 mg cardiac tissue was incubated at 4 °C for varying time periods, and GSH was measured. The data revealed that after 60 min, the concentrations of GSH in extracts were reduced by approximately 35% (Figure 6) possibly due to auto-oxidation shown in Figure 5.

**Figure 7:**
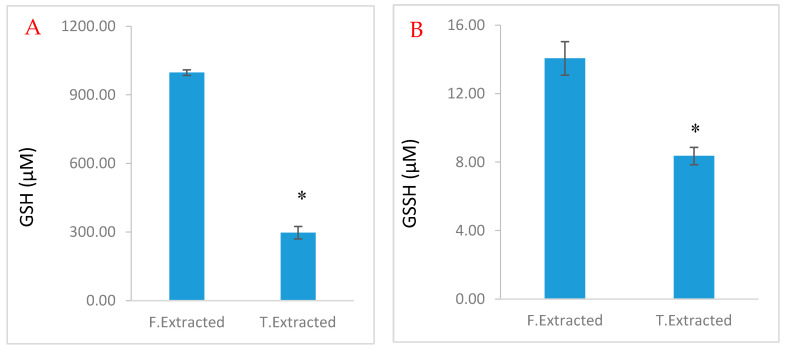
Degradation of blood and serum reduced glutathione (GSH) through different extraction procedures. T extract: Freshly harvested whole blood of serum was snap frozen in liquid nitrogen and stored at −80 °C for 6 weeks. On the day of the experiment, PCA was added to the frozen sample and allowed to thaw, thus extraction and thawing occurred simultaneously; F-extract: freshly harvested whole blood of serum was subjected to PCA extraction and the supernatant stored at −80 °C for 6 weeks. On the day of the experiment, the supernatant was thawed and used for the assay. (**A**) was subjected through GSH protocol; (**B**) was subjected through GSSG protocol. Data are presented as mean ± SEM (*n* = 4, * *p* < 0.05). Data are presented as mean ± SEM (*n* = 4).

**Figure 8:**
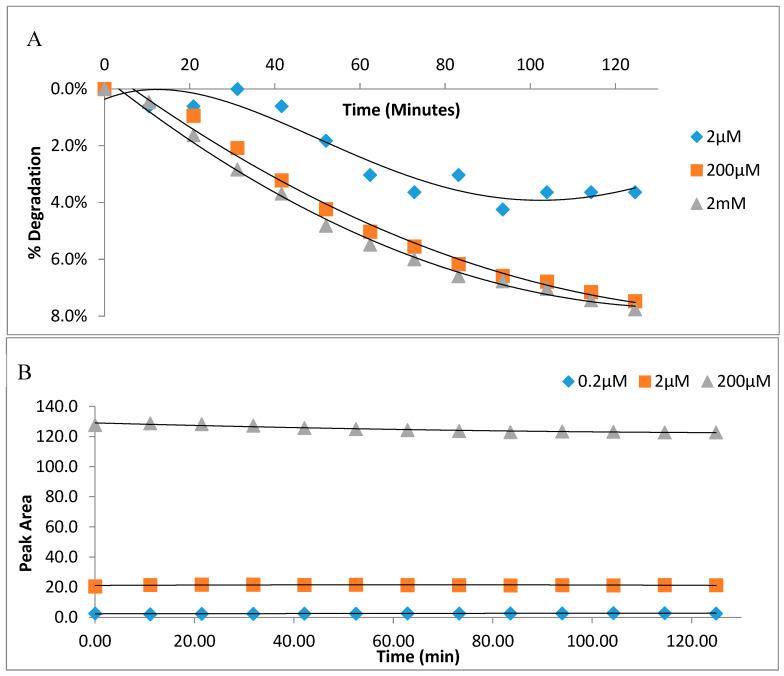
(**A**,**B**): Stability of glutathione-OPA adduct. The time-dependent (over 120 min) degradation at 40 °C of reduced glutathione (GSH) (**A**) and oxidised glutathione (GSSG) (**B**) after derivatisation at three concentrations: 2 μm, 200 μm and 2 mM (**A**). Data are presented as means (*n* = 2).

Unlike powdered frozen tissue, frozen whole blood or serum requires thawing which could increase vulnerability to the auto-oxidation or degradation thus far established. Frozen whole blood or serum extracted (with 5% PCA) simultaneously with thawing (T. Extract) on the day of analysis preserved endogenous GSH (Figure 7A,B). By contrast, when supernatant of PCA extract snap frozen in liquid nitrogen (F. Extract), stored at −80 °C for 6 weeks was used, it resulted in a greater loss of endogenous GSH (Figure 7A,B). The GSH-OPA adduct was stable, degraded in a concentration-dependent manner and retained 96% fluorescence at 120 min (Figure 8A); adduct from GSSG has greater stability (Figure 8B).

The regression analysis (Table 1) of standard curve over the range 0.1 to 4000 µM GSH indicated that the method gave a linear signal for GSH with r^2^ value close to 1 (*p* < 0.001).

**Figure 9:**
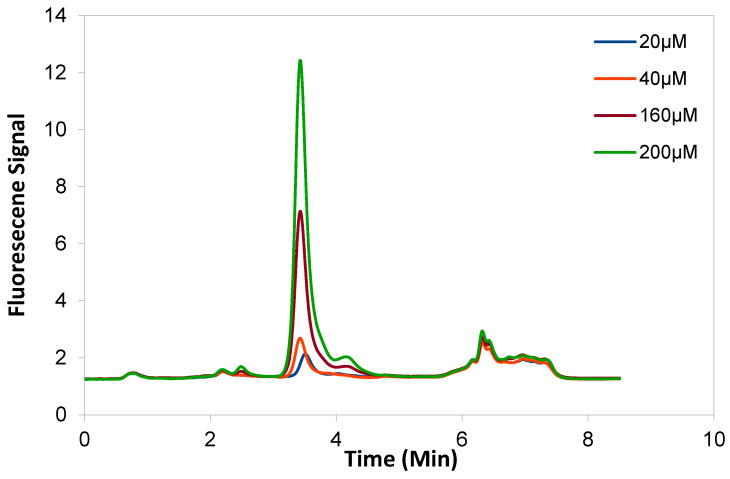
Overlaid chromatograms of the GSH standards at a wavelength of 450 nm.

**Figure 10:**
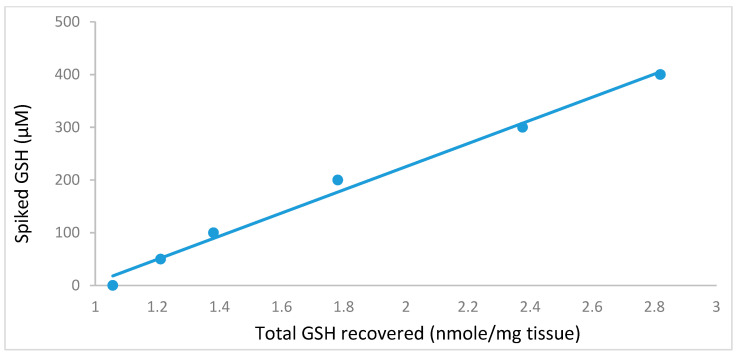
The total reduced glutathione (GSH) recovered from 200 mg of liver tissue spiked with GSH (0–400 μM) prior to extraction (*n* = 3).

The limit of detection (LOD: lowest amount detectable but not necessarily quantifiable) and the limit of quantification (LOQ: lowest amount quantitatively detectable with precision and accuracy) were calculated to be 0.34 µM and 1.14 µM respectively from the regression equation. Both LOD and LOQ were tested and verified experimentally. The LOD and LOQ of GSSG method was found to be 0.26 µM and 0.88 µM respectively. Overlapping chromatograms (Figure 9) of GSH standards further supports that the optimised method has satisfactory specificity with asymmetry factors of 0.821 (20 µM). Five liver samples (≈200 mg each) were spiked with either 50, 100, 200, 300, or 400 µM GSH prior to extraction. The total GSH of the extract increased linearly (Figure 10; r^2^ = 0.99, R = 1) with spiked GSH concentration showing consistent recovery of the extraction procedure.

## 3. Discussion

Oxidative stress is prevalent in many diseases including cancer [41], heart failure [42] and chronic kidney disease [43] and worsens with stage of the diseases. Indeed, there is a growing body of evidence that suggests that in addition to being a consequence of these chronic diseases, oxidative stress could also be a key mediator in their progression [44,45,46]. Therefore, modulation of oxidative status could be a potential therapeutic target and thus its measurement is a prognostic indicator of disease activity and response to treatment. The measurement of glutathione status clinically would be of potential benefit in terms of oxidative stress assessment, hence the need for an easy, rapid, sensitive and reliable method. In that regard, the present study developed and optimised various parameters including stability (time and temperature dependent), and precision of glutathione extraction and recovery. There are several methods of measuring glutathione, which include fluorometric and enzymatic [47,48] with greater discrepancies in the data between these methods [49].

McGill and Jaeschke [50] have studied this problem in detail but as yet no definitive solution has been found. Most of the fluorometric methods reported involved extended sample preparation predisposing them to GSH autoxidation [32,49]. The fluorometric method used by Floreani et al. [49] adapted incubation and centrifugation times of 30 and 15 min respectively during sample preparation as opposed to very rapid times used in the enzymatic method. As a result, their data showed a GSSG level five times higher in the fluorometric method relative to enzymatic method. Sample preparation in the present method was rapid, optimised at 5 min PCA precipitation; 5 min high speed (12,000× *g*) centrifugation; and 5–10 min incubation to overcome the limitation of auto-oxidation similar to the report of Paroni et al. [51]. Also, the addition of NEM earlier during sample preparation for GSSG measurement further prevents GSH auto-oxidation. Total of 40 mM NEM was sufficient to achieve this goal. However, it must be recognised that our method employed the hydrolysis of GSSG to GSH which reacts to OPA, thus GSSG may vary from 0.1–1.0% of the level of GSH. While oxidation of a small proportion of the GSH during sample acquisition and work up might cause the reported total to be a few percent less than the actual one, this same conversion may obliterate any vestige of the original GSSG value.

The auto-oxidation of GSH during sample preparation represented the biggest challenge in the assessment of GSH/GSSG. The concentration of GSH in cardiac tissue fell sharply, reaching a nadir of 35% by 1 h and plateaued. That compared well with authentic GSH standard that underwent auto-oxidation to give a 7.5-fold increase in GSSG concentration in the first 1 h. This is in contrast to the report of Kander et al. [29] where GSH was reportedly stable at 4 °C for 24 h. Also, the authors reported a stable metaphosphoric acid extract at −80 °C for at least 3 months. By contrast, we found a rather stable GSH in whole blood or serum at −80 °C for 6 weeks compared to PCA extracts. PCA aided deprotonation and extraction of whole blood and serum was optimal when done simultaneously with thawing, possibly because it accelerated thawing thus limiting sample idling. Aside from auto-oxidation, glutathione was also temperature sensitive, demonstrated by 15% and 67% loss at 25 °C and 50 °C respectively compared to at 4 °C. The low LOD and LOQ confirmed the high sensitivity of the method.

The recovery of the spiked liver samples was consistent at 96.1 ± 3.4–98.6 ± 2.9% and duplicate variation was not statistically significant. With extraction procedure limited to 10–12 min, 10 min incubation during derivatisation and HPLC set to 4 °C, the method overcame the problems of auto-oxidation of GSH and degradation of glutathione-OPA adduct. The gradient mobile phase and flow rate were also optimised to give a suitable system with consistent peaks elution time and acceptable tailing. The method was subsequently used for oxidative stress studies in cardiac and kidney diseases.

## 4. Materials and Methods

### 4.1. Materials

**Figure 11:**
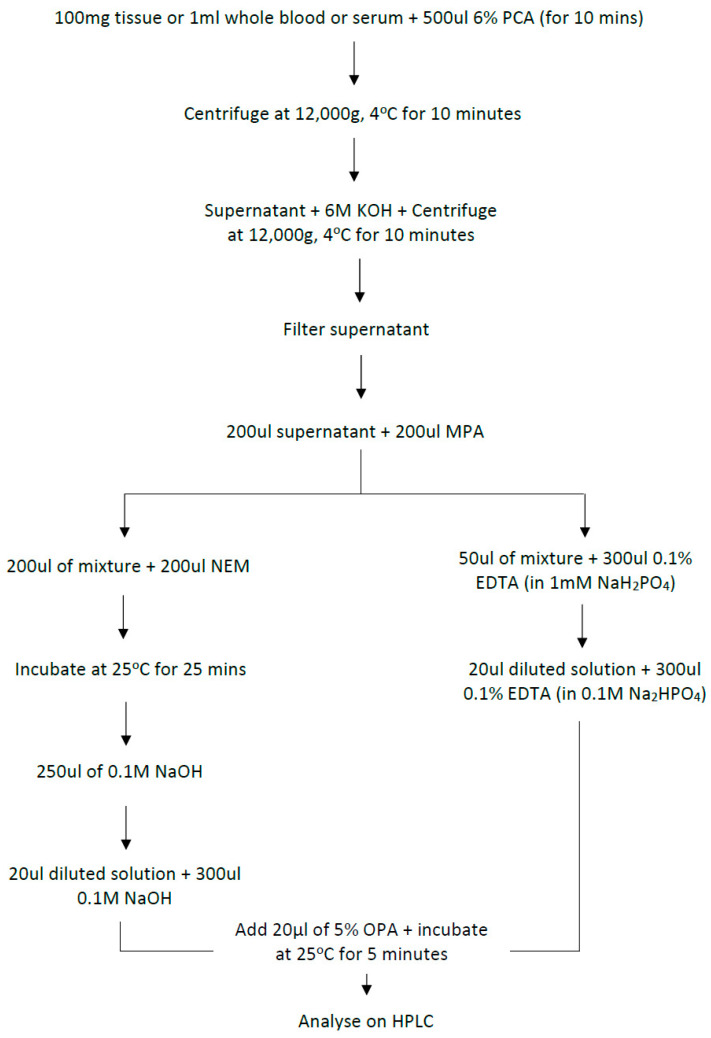
Schematic representation of glutathione assay protocol (every step is done on ice or at 4 °C). High performance liquid chromatography (HPLC); sodium hydroxide (NaOH); *N*-ethylmaleimide (NEM); potassium hydroxide (KOH); ethylenediaminetetraacetic acid (EDTA).

GSH, GSSG, O-pthaldialdehyde (OPA), *N*-ethylmaleimide (NEM), ethylenediaminetetraacetic acid (EDTA) and sodium hydrogen phosphate (NaH_2_PHO_4_) were purchased from (Sigma-Aldrich, Poole, UK). HLPC-grade acetonitrile and methanol were obtained from Fisher Scientific (Leicester, UK) and used without further purification. Perchloric acid (PCA) was obtained from VWR International (Loughborough, England). Chromacol autosampler vials (300 µL) were obtained from Thermo Scientific (Langerwehe, Germany). Chromatographic-grade water was generated from Milli-Q Advantage System^TM^ (Millipore, Watford, UK) and filtered through Whatman Filter Paper (Fisher Scientific, Loughborough, UK). The NaH_2_PHO_4_ mobile phase solvent was degassed and filtered through a 0.45-µm Whatman filter paper. GSH and GSSG standard solutions were prepared fresh each day. O-pthaldialdehyde (OPA) solution in methanol was prepared every 4 hrs and NEM was prepared just before use. A summary of the whole procedure is detailed in Figure 11.

### 4.2. Method Optimisation

Varying amounts of O-pthaldialdehyde (OPA) reagent (1–10%, *v*/*v*) (Sigma-Aldrich, Poole, UK) were titrated with 2 mM GSH to determine the optimal reactivity (product yield). The incubation time for the OPA-glutathione reaction was optimised over 40 min to avoid unnecessary degradation at 25 °C. Total of 40, 80 and 120 mM *N*-ethylmaleimide (NEM) were used to measure GSSG in (a) 20 µM GSSG containing 200 µM GSH (b) 200 µM GSSG containing 2000 µM GSH. The time at which NEM was added (0–60 min) and temperature (at 4 °C, 25 °C and 50 °C) dependent stability of GSH were evaluated by assessing the autoxidation of 200 µM and 2000 µM GSH to GSSG. The stability of pure GSH was compared to that of GSH extracted from 200 mg cardiac tissue to determine the appropriate time for NEM addition during sample preparation.

Thawed perchloric acid (PCA) extracted samples (frozen whole blood or serum (from −80 °C)) and freshly collected whole blood or serum samples were studied to determine the impact of thawing on the determine degradation loss of glutathione.

To examine the stability of total glutathione after derivatisation, GSH (2 µM, 0.2 mM and 2 mM) and GSSG (0.2, 2 and 20 µM) adducts with O-pthaldialdehyde were analysed over 120 min.

### 4.3. Sample Collection and Preparation

All procedures relevant to animals used in this study were carried out in accordance with the UK Animals (Scientific Procedure) Act 1986 and were approved by the University of Hull Ethical Review Process (No. PPL 70/7966). Cardiac, skeletal, liver and kidney tissues from male Sprague-Dawley rats were washed for excess blood, freeze clamped with Wollenberger tong cooled in liquid nitrogen and stored at −80 °C. Tissues were subsequently weighed and powdered in liquid nitrogen according to Seymour et al. [40]. At time of tissue harvesting, blood samples were taken, and a portion centrifuged at 4 °C. The serum was obtained and whole blood was snap frozen in liquid nitrogen and stored at −80 °C for future glutathione analysis.

Powdered tissues, whole blood and serum were subjected to 6% PCA extraction [51] and neutralised with 6 M potassium hydroxide (KOH). The resultant supernatant was filtered through 0.45 µm Millex syringe-driven Filter unit (Merck KGaA, Darmstadt, Germany) for glutathione analysis.

### 4.4. Sample Derivatisation

Sample derivatisation was carried out according to a modification of method of Kander et al. [31]. In addition, protein crash with 200 μL 10% metaphosphoric acid (MPA) added to the filtered sample supernatant at 4 °C was performed to minimise HPLC fouling.

For GSH assay, extracted samples were initially diluted seven-fold with 0.1% ethylenediaminetetraacetic acid (EDTA) in 0.1 M sodium hydrogen phosphate (Na_2_HPO_4_) (pH at 8.0) and 20 µL diluted solution mixed in a glass vial with 300 µL 0.1% EDTA in 0.1 M Na_2_HPO_4_ (pH at 8.0)) to give GSH assay mixture. For GSSG assay, 100 µL extracted sample was added to 100 µL of 40 mM *N*-ethylmaleimide (NEM) (1:1 ratio), incubated at 25 °C for 25 min and mixed with 250 µL 0.1 M sodium hydroxide (NaOH). Of the resultant solution, 20 µL was added to a glass vial containing 300 µL of 0.1 M NaOH. Total of 20 µL 10% O-pthaldialdehyde (OPA) in methanol was added to each assay mixture, vials were capped, the resulting reaction mixture incubated at 25 °C for 5 min in the dark and analysed using high performance liquid chromatography (HPLC) (Merck KGaA, Darmstadt, Germany).

### 4.5. Standard Curves

Glutathione calibration curves were prepared using GSH and GSSG standards (Sigma-Aldrich, Poole, UK). Stocks of 20 mM GSH and GSSG were prepared individually in 0.1 mM hydrochloric acid (HCl). Working stocks of GSH (5-fold and 50-fold dilutions) were prepared and standard curves of GSH, covering the range of 0–20 µM (for analysis of serum), 0–2000 µM (for analysis of whole blood) and 0–500 µM (for analysis of tissue) were generated. For GSSG, 0–400 µM (for analysis of whole blood) and 0–20 µM (for analysis of tissue and serum) standard curves were used. Standards were analysed by HPLC. Repeat injections for each standard curve (5 separate injections) were used to evaluate the linearity and range of the method. Accuracy and recovery of GSH and GSSG were determined by adding increasing amount (0–400 µM) of each standard to cardiac tissue—GSSG standard was pre-treated with NEM prior to spiking. Authentic GSSG standard from Sigma was subjected to NEM treatment followed by hydrolysis under basic condition followed by reaction with OPA. Only one fluorescent product of GSH-OPA adduct was detected whose peak area changes linearly with changes in concentration. Spiking of known amount of tissue (hepatic or cardiac) with serial concentration of authentic GSSG prior to extraction and derivatisation produced a linear curve. These confirmed that the intensity of the peak of GSH-OPA adduct (from the GSSG protocol) was directly proportional to the amount GSSG. In addition, no interfering peak was detected.

### 4.6. Glutathione Assay

**Figure 12:**
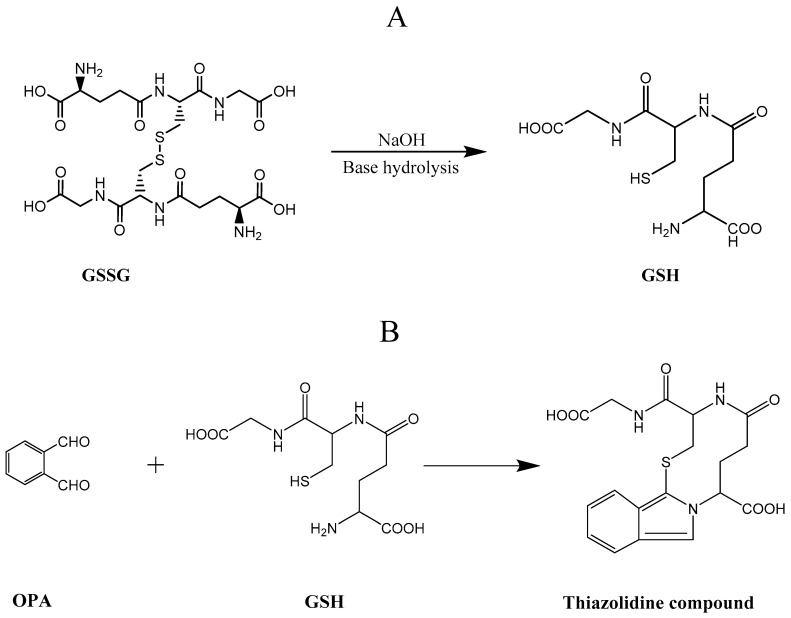
with O-pthaldialdehyde (OPA) glutathione reaction. (**A**) Oxidised glutathione (GSSG) is hydrolysed under condition of 0.1 M Sodium hydroxide (NaOH) to give reduced glutathione (GSH) (**B**) hetero-bifunctional OPA reaction with GSH.

A mixture of OPA and GSH at basic pH and optimal temperature (25 °C) generate a fluorescent thiol adduct via heterobifunctional OPA reaction with an excitation wavelength of 350 nm and emission at 450 nm. GSSG is initially hydrolysed to GSH which reacts with OPA as shown in Figure 12.

Chromatographic analysis of the GSH-OPA abduct was performed using reverse-phase chromatography on Agilent 1200 HPLC (Agilent Technologies, Stockport, UK) with G1311a Quaternary pump, G1367a Autosampler (5 μL injection), G1316a Column Compartment (37 °C) G1321B Fluorescent light detector, fitted with Phenomenex Gemini C18 100X2 mm and an additional guard column (Phenomenex, Macclesfield, UK) and a stepwise solvent gradient of 25 mM NaH_2_PO_4_ (pH 6.0) and acetonitrile as the mobile phase. The column was initially hydrated with 0.5% acetonitrile and 99.5% of the phosphate buffer (NaH_2_PO_4_) at a linear rate (Table 2) from 0.4 mL/min to 0.6 mL/min over 4 min, increased to 2% acetonitrile for 0.5 min at 1.25 mL/min and then to at 1.5 mL/min over 1.5 min, then changed linearly to 65% acetonitrile over 0.9 min and finally returned to 0.5% acetonitrile and 99.5% of the phosphate buffer at 1.5 mL/min for 2 min equilibration. Data analysis was performed on Agilent Chemstation software and peaks quantified using GSH and GSSG standard curves.

### 4.7. Statistical Analysis

Difference between samples analysed were calculated using student T test and linearity and correlation of standard curves by regression analysis using SPSS (IBM, New York, NY, USA). Data are presented as mean ± standard error of the mean (SEM).

## 5. Conclusions

The developed and optimised method has been tested repeatedly, validated and found to be suitable for the determination of both GSH and GSSG and hence their ratio as an index of oxidant status of a biological system. The validation assessed the linearity, range, specificity, LOD, LOQ, accuracy, precision and analyte recovery of the HPLC method. All these parameters were found to be consistent and within the acceptable range. The current method showed great potentials and applications in future clinical studies [5].

## Figures and Tables

**Table 1:** Linearity and prediction power of the glutathione method. X and Y are the glutathione concentration and detector signal, respectively (GSSG: *p* < 0.001).

Regression Analysis for the Range of GSH and GSSG Standard Curves		
Linearity range	1.2 µM–4 mM	GSH
	1.0 µM–0.4 mM	GSSG
Slope	0.413	GSH
	1.27	GSSG
Intercept	20.16	GSH
	−8.78	GSSG
Regression equation	Y = 0.413x + 21.16	GSH
	Y = 1.27x − 8.78	GSSG
Coefficient of Determination (r^2^)	99.6%	GSH
	99.6%	GSSG
Coefficient of correlation (R)	1.00	GSH
	1.00	GSSG
Limit of detection (LOD)	0.34 µM	GSH
	0.26 µM	GSSG
Limit of quantification (LOQ)	1.14 µM	GSH
	0.88 µM	GSSG

**Table 2:** Chromatography conditions.

Time (min)	0	4.0	4.5	6.0	6.5	6.51	8.5
%A (phosphate buffer)	99.5	98	98	65	65	99.5	99.5
%B (ACN)	0.5	2	2	35	35	0.5	0.5
Flow Rate mL/min	0.4	0.6	1.25	1.5	1.5	1.5	1.5
